# Regional distribution of the MTHFR C677T polymorphism in Chinese females

**DOI:** 10.3389/fgene.2023.1139124

**Published:** 2023-04-21

**Authors:** Hua Lin, Changxi Liao, Rujing Zhang

**Affiliations:** ^1^ Affiliated Hospital of Putian University, Putian University, Putian, China; ^2^ Key Laboratory of Medical Microecology (Putian University), Fujian Province University, Putian University, Putian, China

**Keywords:** methylenetetrahydrofolate reductase, female, genetic polymorphism, C677T, Chinese

## Abstract

**Objective:** For analyzing the distribution characteristics of MTHFR C677T polymorphism in Chinese females in order to provide information for reducing birth defects and formulating public health policies to prevent congenital malformations.

**Methods:** Literature search in the last 6 years on “MTHFR C677T,” “polymorphism” and “methylene tetrahydrofolate reductase.” The included literature provides the MTHFR C677T frequency in healthy females in the corresponding regions. The data were grouped by the national administrative region as a unit to obtain the distribution information of the MTHFR C677T and alleles in the female population in every province, municipality or autonomous region. This was done for analyzing the overall distribution of the MTHFR C677T allele and the geographical distribution of pregnancy complications.

**Results:** A total of 126 studies were included, covering five autonomous areas, four municipalities directly under the Central Government, as well as 22 provinces (except Taiwan Province) in China. MTHFR C677T polymorphism data of 27 groups of Chinese Han women and 31 groups of other Chinese females were obtained, and the chi-square test revealed notable inter-group differences (*p* = 0.000). The TT genotype and T allele of MTHFR C677T accounted for 18.2% (4.7%–38.3%) and 40.3% (19.7%–61.4%) of the Chinese female population, respectively, with a significant north-south difference. Chinese females had a consistent frequency of the T allele with the geographical distribution of pregnancy complications such as recurrent abortion and preeclampsia.

**Conclusion:** With a obvious geographical gradient, the MTHFR C677T polymorphism distribution in Chinese females is consistent with the geographical distribution of multiple pregnancy complications, and the risk assessment for it might be included in primary prevention for birth defects.

## 1 Introduction

As an essential enzyme in folic acid (FA) metabolism, methylenetetrahydrofolate reductase (MTHFR) is able to catalyze the transformation of 5,10-methylene-THF into 5-MTHF, the main methyl donor for homocysteine to be transformed into methionine, DNA and RNA. C677T and A1298C are two frequently-seen polymorphisms of MTHFR. The polymorphism C677T is the most common in South America as well as Asia. The change in C677T replaces the alanine at the 222nd amino acid site with valine, which will lead to greatly reduced enzyme stability and is the most frequently-seen genetic cause of hyperhomocysteinemia. Polymorphisms of A1298C are common in Europe and North America and are rare in Asia (1%–4%). This change causes glutamate at the 249th position to be replaced by alanine, which affects the MTHFR activity and thermal stability, also leading to hyperhomocysteinemia (Chango et al., 2000; Al Hammouri et al., 2022).

Homocysteine concentrations rise after the enzyme’s homeostatic action is disrupted, leading to a series of pathological disorders that develop one after the other. Hyperhomocysteinemia has been demonstrated to raise the pathogenic risk of cardiovascular and neurological illness (Tinelli et al., 2019; Al Mutairi, 2020). FA, as one of the key micronutrients during pregnancy, and homocysteine accumulation can have harmful effects on maternal health and fetal growth (Kanasaki and Kumagai, 2021). Studies have shown that hyperhomocysteinemia is associated with many complications related to pregnancy, including preeclampsia, recurrent abortion, fetal growth retardation, gestational diabetes mellitus (TM), placental abruption, premature delivery, and so on (Nwogu et al., 2020; Dai et al., 2021). If there is a homozygous mutation of the MTHFR C677T TT genotype in pregnant females, it is generally accompanied by a lack of FA, and the serum homocysteine level may increase, which will greatly enhance the probability of congenital anomalies in the offspring. Additionally, the T allele in C677T is highly associated with maternal TG levels in pre-eclampsia, a risk factor for severe eclampsia and hypertension in pregnancy (Parthasarathy et al., 2023).

China has a vast territory, a large population, diversified ecology and diet, and the MTHFR C677T polymorphism is significantly different in various geographical regions and among diverse ethnic groups, a result of long-term interactions between heredity and the environment. Clarifying the genetic polymorphisms and distribution of MTHFR C677T among women in different regions and understanding the differences in FA utilization among women in China will help the country formulate public health policies and related research in the field of genetic diseases. In this study, the polymorphism distribution characteristics of the MTHFR C677T gene in healthy women from four municipalities, five autonomous regions, and 22 provinces in China were summarized and analyzed. A vector map was adopted for describing the overall distribution pattern of the MTHFR C677T T allele in Chinese females. The gene polymorphisms showed a regional distribution pattern, and the risk assessment could be formulated according to local conditions, thus, primary prevention measures for congenital anomalies could be developed.

## 2 Materials and methods

### 2.1 Document retrieval

The literature on the MTHFR C677T polymorphism in women from different regions in China reported over the past 6 years was retrieved from the MEDLINE, CNKI, Wanfang, and VIP databases. Chinese keywords “MTHFR C677T,” “Methylenetetrahydrofolate reductase,” “Polymorphism,” English keywords “MTHFR C677T,” “Polymorphism” were used in a combined review of the references cited in various articles. The search deadline was December 2021.

### 2.2 Literature inclusion and exclusion criteria

Inclusion criteria: ① Published within the last 6 years (2016–2021); ② The subjects were Chinese females; ③ Reports on the frequency of MTHFR C677T in this region; ④ The data were consistent with Hardy-Weinberg genetic equilibrium. Exclusion criteria: ① Incomplete data (Al Hammouri et al., 2022); Repeated published studies (literature with publications based on the same population sample) (Tinelli et al., 2019); The design of the included samples, such as cohort studies or case‒control studies (Al Mutairi, 2020); Non-original studies such as reviews or meta-analyses.

### 2.3 Literature screening and data extraction

In the initial screening of the literature, irrelevant literature was excluded in light of the title and abstract content. Then, based on the inclusion/exclusion criteria, the full text was further screened to determine whether to include it. The screening process was independently conducted by two reviewers, and the screening results were cross-checked until an agreement was reached. The extracted data included basic information such as region, a detection unit, ethnicity, age, sample size, first author and publication year, as well as the MTHFR C677T genotyping method and frequency of each genotype.

### 2.4 Statistical analysis of the MTHFR C677T gene distribution

The Hardy-Weinberg genetic balance test was performed on the data from each region, and *p* > 0.05 indicated that the data were representative of the regional population; otherwise, they were excluded. The data of each region were combined according to the national administrative level, and the female MTHFR C677T polymorphism distribution in each municipality, autonomous region, or province was obtained. The inter-group comparison of genotype or allele was conducted via *χ* ^2^ tests, and *p* < 0.05 indicated a notable difference. Vector mapping was used to map the T allele distribution at the MTHFR C677T locus in Chinese Han women and Chinese women of other ethnicities.

### 2.5 Statistical analysis of the geographical distribution of diseases

According to the China Health Statistical Yearbook 2021 and the original data of relevant literature investigations, maternal or fetal deaths caused by diseases of pregnancy such as hypertension and preeclampsia in various municipalities, autonomous regions or provinces were counted, and the relevant distribution information was drawn using vector maps.

## 3 Results

### 3.1 Literature screening and results

**FIGURE 1:**
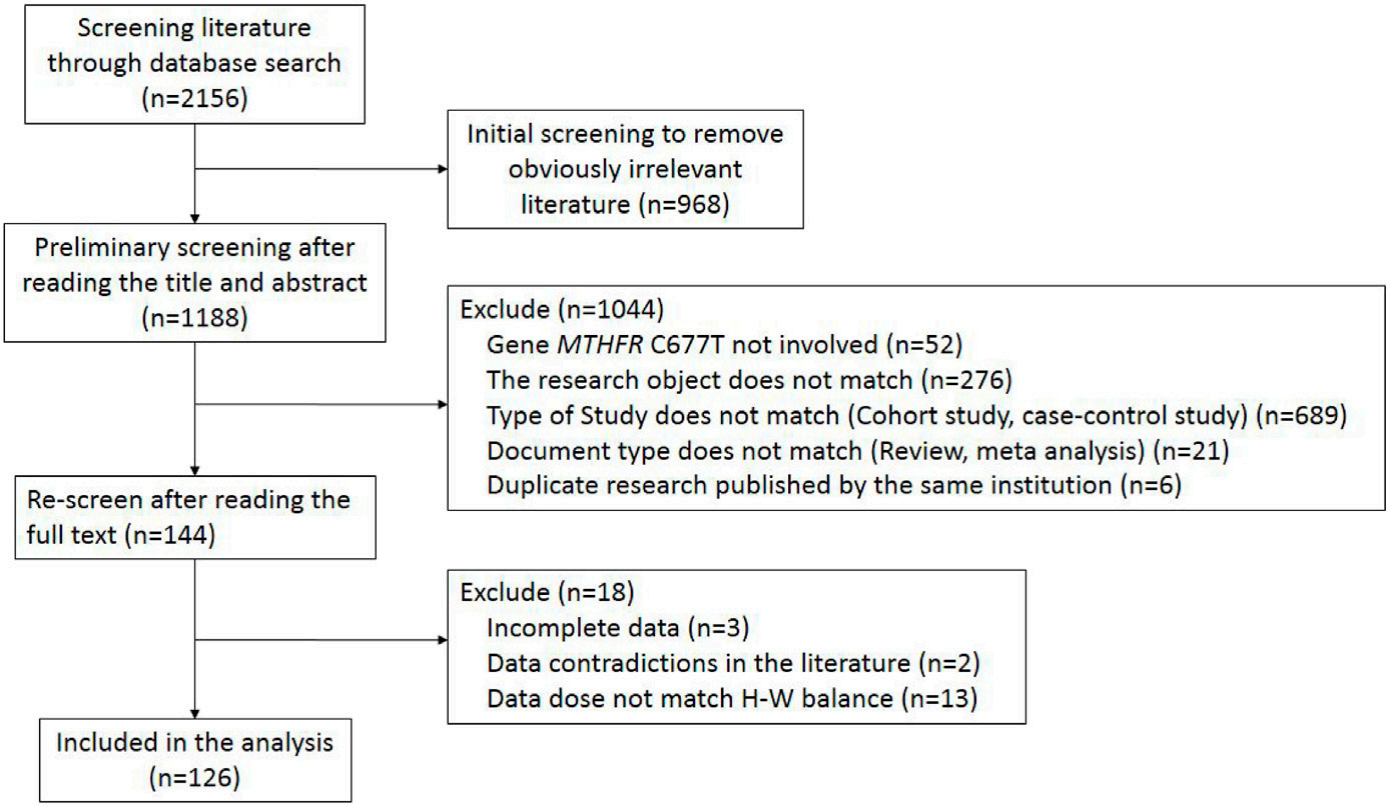
The flowchart of literature screening process.

According to the literature retrieval method set by the study, a total of 2,156 references were obtained. After eliminating 2030 non-standard studies by step-by-step screening, 126 studies were finally enrolled. The specific screening process is summarized in Figure 1.

### 3.2 Basic characteristics

Among the 126 articles meeting the inclusion criteria, the MTHFR C677T polymorphism data of women from four municipalities, five autonomous regions and 22 provinces (except Taiwan Province) in China were covered, of which 90 articles had specific ethnic groups. Among all of the research participants providing data, there were 144,996 Han women and 24,700 women from ethnic minorities.

### 3.3 MTHFR C677T gene distribution in Chinese Han women

**FIGURE 2:**
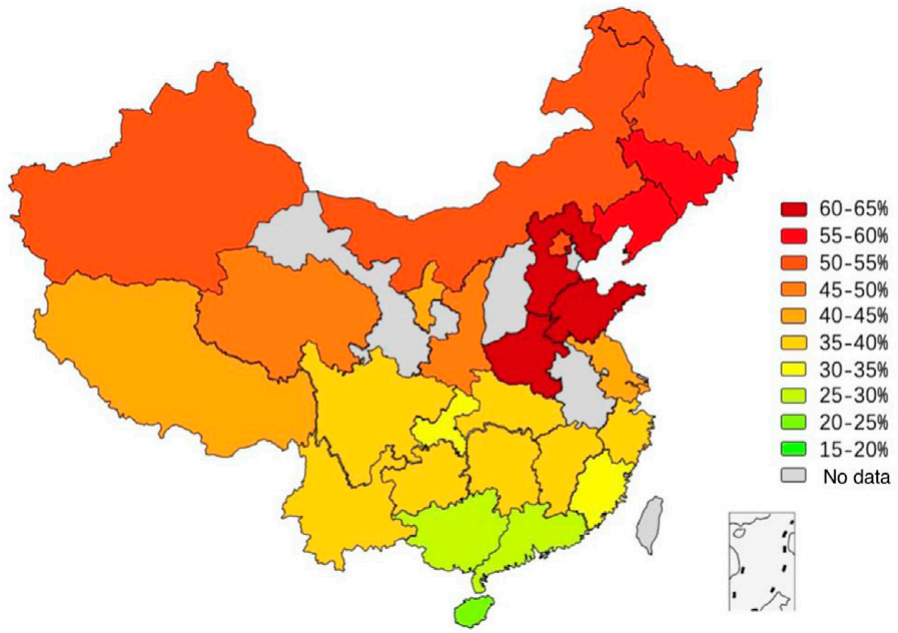
Vector map showing the T allele distribution of MTHFR C677T in Han ethnicity women.

The MTHFR C677T gene distribution data of Chinese Han women reported in the past 6 years were combined according to the national first-level administrative regions (municipalities, autonomous regions and provinces), and 27 groups of Han women data were obtained (Supplementary Table S1). The inter-group genotype difference was notable (*p* = 0.000). Except for Han females in Chongqing, Fujian, Guangdong, Guangxi and Hainan, the wildtype CC genotype was dominant, and the heterozygous CT genotype was dominant in the remaining 22 groups of data. By mapping the vector map of the T mutation gene, we can intuitively reflect the distribution pattern of the MTHFR C677T locus in Han females in various regions (Figure 2). Among them, the T allele frequency in Shandong, Henan and Hebei was the highest (62.8%, 62.4% and 61.4%, respectively), and in other regions, it generally increased from south to north. Chinese Han women had total frequencies of 19.2% and 41.4% in the TT genotype and T allele, respectively.

### 3.4 MTHFR C677T gene distribution in Chinese females

**FIGURE 3:**
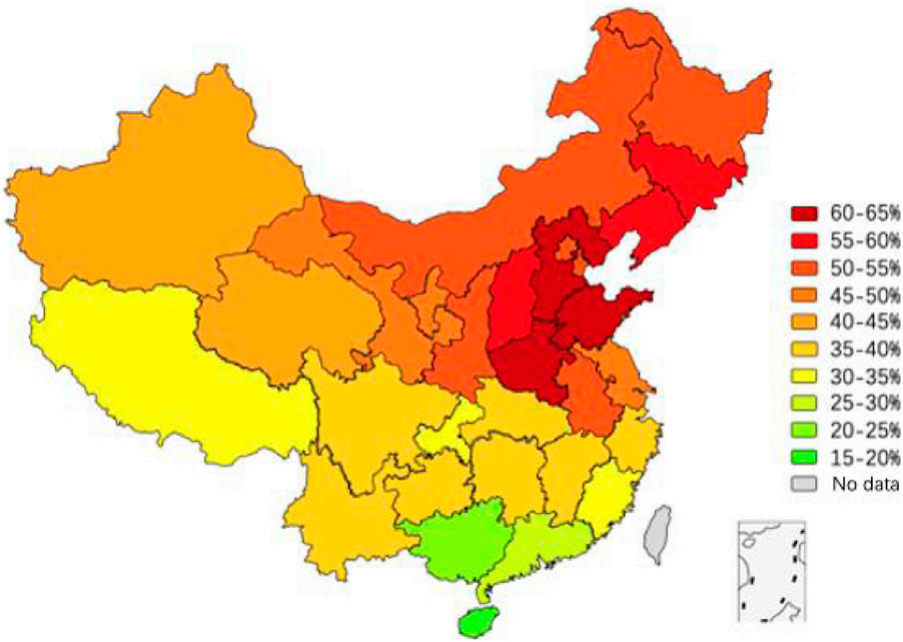
Vector map showing the T allele distribution of MTHFR C677T in Chinese women.

The MTHFR C677T gene distribution data of Chinese females reported in the past 6 years were combined according to the national first-level administrative regions (municipalities, autonomous regions and provinces), and 31 groups of data were obtained (Supplementary Table S1). The inter-group genotype difference was notable (*p* = 0.000). Compared with the data of Han women, the T allele frequency in Shanxi, Tianjin, Anhui as well as Gansu, which are not clear about the ethnic groups in the literature, was increased. The TT genotype and T allele of MTHFR C677T accounted for 4.7%–38.3% and 19.7%–61.4% in the female population, respectively, with a notable disparity between the northern and southern individuals. The T gene frequency in Shandong and Henan ranked in the top three in China (61.2%, 60.4%, 61.4%) with Hebei after supplementing the data of ethnic women in the literature. In addition, after the data of ethnic minority groups were supplemented in 12 regions of Liaoning, Jilin, Inner Mongolia, Qinghai, Xinjiang, Hubei, Guizhou, Yunnan, Hunan, Tibet, Guangxi and Hainan, the frequency of T gene mutation decreased to varying degrees, among which the allele frequency differences between Xinjiang, Guizhou, Yunnan, Tibet, Guangxi and Hainan (*p* = 0.000, 0.003, 0.000, 0.000 and 0.000) were statistically significant. The dominant genotype in Tibet was the CC type (while the dominant genotype in Han women was the CT type). By comparing the distribution vector map of the T gene between Han women and other Chinese females, the variation in the T gene frequency can be more intuitively presented (Figure 3). The frequency of T mutation genes in the female population showed a trend of a gradual increase from south to north and from west to east, and TT genotype and T allele had total frequencies of 18.2% and 40.3%, respectively.

### 3.5 Regional distribution of maternal mortality caused by pregnancy-induced hypertension in Chinese females

According to the statistical analysis of the data in the 2021 China Health Statistics Yearbook, a summary table of maternal death causes in 2020 (Supplementary Table S2) was obtained. The main factors of maternal death caused by gestational hypertension and medical complications have certain regional distribution differences.

**FIGURE 4:**
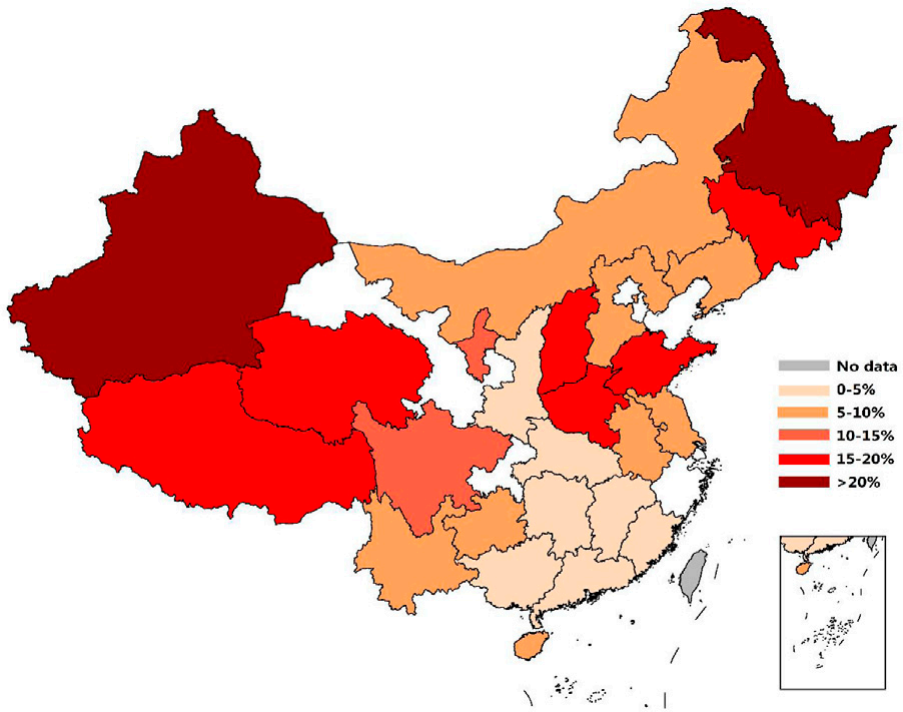
Vector map showing the distribution of gestational hypertension-caused deaths in Chinese women in 2020.

Among them, the proportion of pregnancy-induced hypertension in maternal mortality is comparatively high in the Heilongjiang, Xinjiang, Jilin, Shanxi and Henan provinces and comparatively low in the Zhejiang, Chongqing, Guangdong, Guangxi and Fujian provinces (Zhu et al., 2021a). Overall, the proportion of maternal mortality caused by pregnancy-induced hypertension gradually increased from south to north, which is similar to the MTHFR C677T gene distribution in Chinese Han women (Figure 4).

**FIGURE 5:**
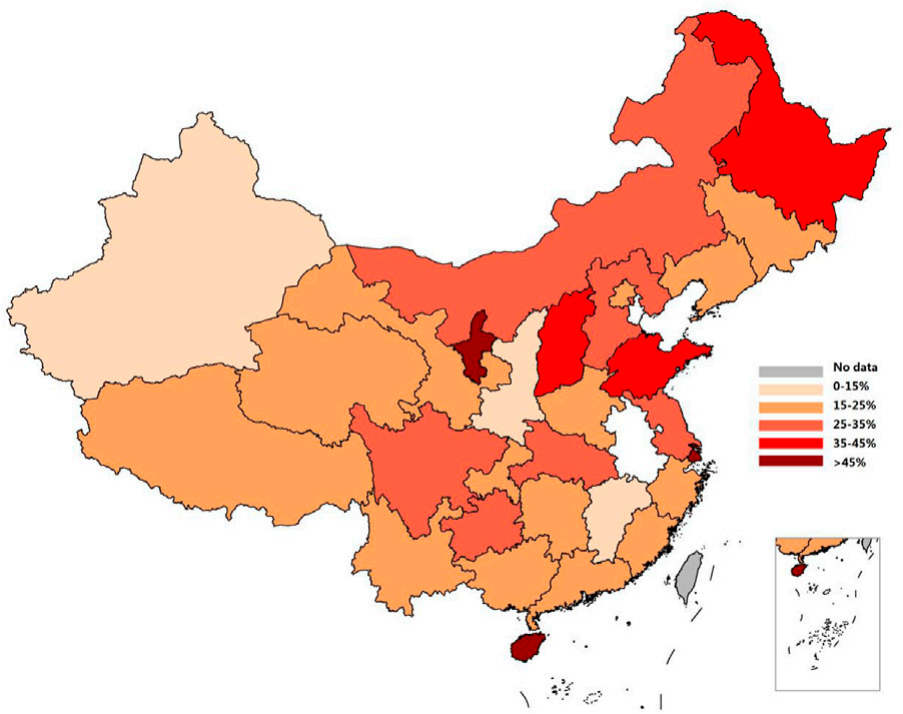
Vector map showing the distribution of medical comorbidities causing death in Chinese individuals in 2020.

In terms of maternal mortality caused by medical complications, except for Hainan, maternal mortality caused by medical complications in most southern regions is relatively low, while maternal mortality caused by general medical complications in eastern and northeastern China was relatively high (Figure 5).

### 3.6 Geographical distribution of neonatal stillbirth causes in China

A cross-sectional survey of causes of neonatal death with a gestational age of 24 weeks and above in 96 hospitals from 24 provinces and cities in 2015-2016 showed that older mothers, chronic hypertension, TM and preeclampsia were risk factors for neonatal death. In the different regions, there were also some differences in the severity of the threat of neonatal death (Table 1). Overall, maternal chronic hypertension, pre-pregnancy obesity, preeclampsia and eclampsia were important causes of neonatal death. Among the 75,132 newborns included in the analysis, chronic hypertension and pre-pregnancy obesity were higher in the east and north. Preeclampsia and eclampsia were important causes of neonatal death in the northeast, north and central regions.

**TABLE 1:** Weighted ratio of stillbirths with risk factors via Chinese geographical region.

Area	Preeclampsia/eclampsia N (%)	Chronic hypertension N (%)	Pre-pregnant obesity N (%)
East	3,449 (8.9)	3,110 (8.0)	4,578 (11.8)
Northeast	356 (9.1)	0 (0.0)	71 (1.8)
Northwest	553 (7.3)	0 (0.0)	254 (3.4)
Southwest	631 (5.9)	237 (2.2)	233 (2.2)
North	1,204 (25.9)	458 (9.8)	646 (13.9)
Central	4,591 (22.7)	13 (0.1)	138 (0.7)
South	217 (1.8)	62 (0.5)	164 (1.3)

## 4 Discussion

The correlation of MTHFR C677T polymorphism with associated maladies has always been a research hotspot. A number of retrospective studies have found that the CT heterozygous mutation of the MTHFR gene retains approximately 60% of its enzymatic activity, while the homozygous TT mutation retains only 10%–20% of its enzymatic activity (Dai et al., 2021). However, the vitamin D and niacin levels in the TT genotype are significantly lower, and the levels of homocysteine are significantly higher, and in contrast to the CT genotype, the TT genotype is linked to a higher risk of preterm birth, recurrent abortion, and offspring with Down syndrome (Rai et al., 2014; Fang et al., 2018; Zarfeshan Fard et al., 2019; Ota et al., 2020). In addition, the MTHFR C677T mutation, accompanied by increased blood homocysteine concentrations, results in a higher risk of various adverse pregnancy outcomes and offspring with congenital cardiovascular disease, neural tube defects (NTDs), or cleft lip and palate (Van Der Put et al., 2001; Wilcken et al., 2003; Pan et al., 2015; Zhang et al., 2018).

This study summarized and analyzed the MTHFR C677T polymorphism in Chinese females, covering 5 autonomous areas, 4 municipalities directly under the Central Government, as well as 22 provinces (except Taiwan Province) in China. Both the homozygous TT genotype of MTHFR C677T and the frequency of the T mutation gene showed a trend of increasing from south to north in both the Han population and the rest of the female population, contrary to that in Europe and North America, which is consistent with the increasing incidence of NTDs from Chinese southeast to northwest. The correlation of MTHFR C677T polymorphism with offspring NTDs has been verified (Pepe et al., 1998; Ren et al., 2007). In addition, a number of reports have shown that preeclampsia, gestational hypertension and preterm delivery are linked to the MTHFR C677T polymorphism. According to relevant intervention studies, FA supplementation (FAS) is able to significantly lower the incidence of preterm delivery, gestational hypertension and TM in pregnant females and lower the risk of light birth weight as well as small fetuses for gestational age. (Li et al., 2015; Li et al., 2017; Zhu et al., 2021a; Zhu et al., 2021b; Lyu et al., 2021). Hyperhomocysteinemia causes vascular and metabolic abnormalities, and when the immune interaction between the fetus and the present is defective, there are abnormalities in the spiral arteries of the uterus, and the blood oxygen and nutritional status supplied to the fetus is affected, and the present often suffers from hypertension to increase blood flow (Stamler and Slivka, 1996; Parthasarathy et al., 2023). FA supplementation helps to improve metabolic abnormalities and promote homocysteine conversion, thereby reducing the incidence of preterm labour and gestational hypertension in pregnant women and reducing the risk to the foetus (Li et al., 2015; Shen et al., 2019). Consistent with previous studies, this study found that maternal or neonatal deaths caused by pregnancy-related diseases such as pregnancy-related hypertension, preeclampsia and eclampsia tended to increase from south to north and from west to east by analyzing the causes of maternal death and the regional distribution of neonatal stillbirth in China. The regional distribution trend of the T allele frequency was similar to that of the pregnancy-related conditions.

The distribution of gene polymorphisms is influenced by heredity, population migration, diet and environment. Research has revealed that the frequency of MTHFR C677T in Han women is higher or significantly higher than that of local minorities (Yang et al., 2013; Cui et al., 2015). Therefore, in our study, compared with the data of Han women, the overall T-allele frequency in women in most regions containing ethnic minority samples decreased to varying degrees, except in the Ningxia region, which was thought to be biased due to the inclusion of a large proportion of unspecified ethnic groups in the data from this region. In our study, the MTHFR C677T frequency in Beijing and Tianjin, which are surrounded by Hebei Province, was lower than that in Hebei Province, which is thought to be related to population migration, and the corresponding proportion of maternal deaths caused by gestational hypertension and medical complications was also lower. Given the differences in eating habits, the FA intake is lower than in the southern or northern China populations (Cui et al., 2021; Li et al., 2021). However, the frequency of homozygous mutations in the TT genotype in northern people is greater than in southern people, which reflects the complexity of gene-environment interaction, and also suggests that prompt state for a particular crowd formulates relevant preventive measures (e.g., supplemental FA) for specific populations is necessary. The influence of perinatal FAS on the prevention of NTDs in offspring has been well established; however, its effect is affected by the MTHFR C677T polymorphism (Tabatabaei et al., 2022; Parthasarathy et al., 2023). The large difference in the MTHFR gene distribution between the north and the south may be an important reason for the difference in the effectiveness of routine FAS in preventing congenital anomalies (Jiang et al., 2021). Guidelines on FAS during pregnancy for the prevention of NTDs issued by the Chinese Association of Maternal and Child Health Care and guidelines on FAS during pregnancy by the Canadian Association of Gynecologists have both proposed FAS for different MTHFR genotypes to balance the risk of excessive FAS and reduce congenital anomalies at the same time (Group MVSR, 1991; Berry et al., 1999; Hao et al., 2003; Wang et al., 2011; Anderson et al., 2013).

Our study summarized the MTHFR C677T polymorphism distribution in China, which will provide information for population genetics and future gene-disease association studies. However, there are some limitations to this study. First, due to the consideration of collecting only relatively new genetic data, the included literature was limited to publications within the last 6 years, which may result in the loss of early data from some regions. Second, the amount of MTHFR C677T polymorphism data for ethnic minorities in existing reports is too small. Given the differences in this gene polymorphism among different ethnic groups, the statistical results of T mutation genes in regions with wide geographical areas or large ethnic minority populations may be biased. Third, some studies do not make specific distinctions in the ethnic classification of the population, which makes the data for Han women in this paper imperfect. Fourth, the analysis of pregnancy-related diseases by region is flawed because of a lack of regional reporting of the incidence of pregnancy-related diseases and the causes of maternal death. As a result, the relationship between pregnancy-related disorders and the MTHFR C677T polymorphism was not accurate. Even so, this study is a summary analysis with the most comprehensive coverage and the largest sample size to date discussing the MTHFR C677T polymorphism distribution in Chinese females. The regional distribution of pregnancy-related disorders and that of the MTHFR C677T polymorphism verifies the correlation of the polymorphism with pregnancy-related disorders in women and their offspring further.

To sum up, this study shows that the MTHFR C677T polymorphism distribution in Chinese females varies significantly by geographical location, and the trend is similar to the trend of neonatal stillbirths due to pregnancy-related complications. These baseline data can be used for understanding the prevalence of MTHFR polymorphisms in Chinese females and providing human genetic data for national public health policy-making. In addition, the MTHFR C677T project should be carried out to assess the FA metabolism capacity of local women and develop individualized FA supplement measures while improving regional genetic databases to lower the possibility of congenital anomalies among offspring to achieve healthy births and healthy children.

## Data Availability

The original contributions presented in the study are included in the article/Supplementary Material, further inquiries can be directed to the corresponding author.

## References

[B1] Al Hammouri A. Misk R. A. Abumunshar H. Abunejma F. M. Idrees T. S. Abu Arqoub M. (2022). Intrauterine limb ischemia in patient heterozygous for the 677C>T) RS1801133 (polymorphism of methylenetetrahydrofolate reductase MTHR gene. Case Rep. Pediatr. 2022, 2701548. 10.1155/2022/2701548 36313901 PMC9616673

[B2] Al Mutairi F. (2020). Hyperhomocysteinemia: Clinical insights. J. Cent. Nerv. Syst. Dis. 12, 1179573520962230. 10.1177/1179573520962230 33100834 PMC7549175

[B3] Anderson C. A. Beresford S. A. McLerran D. Lampe J. W. Deeb S. Feng Z. (2013). Response of serum and red blood cell folate concentrations to folic acid supplementation depends on methylenetetrahydrofolate reductase C 677 T genotype: Results from a crossover trial. Mol. Nutr. food Res. 57 (4), 637–644. 10.1002/mnfr.201200108 23456769 PMC4132693

[B4] Berry R. J. Li Z. Erickson J. D. Li S. Moore C. A. Wang H. (1999). Prevention of neural-tube defects with folic acid in China. China-U.S. Collaborative project for neural tube defect prevention. N. Engl. J. Med. 341 (20), 1485–1490. 10.1056/NEJM199911113412001 10559448

[B5] Chango A. Boisson F. Barbé F. Quilliot D. Droesch S. Pfister M. (2000). The effect of 677C-->T and 1298A-->C mutations on plasma homocysteine and 5,10-methylenetetrahydrofolate reductase activity in healthy subjects. Br. J. Nutr. 83 (6), 593–596. 10.1017/s0007114500000751 10911766

[B6] Cui H. Lu Y. Ma S. Xue Y. Wang T. Duan G. (2015). Geographical distribution of MTHFR and MTRR gene polymorphisms among the Han women in Zhengzhou city. Zhong Nan Da Xue Xue Bao Yi Xue Ban. 40 (7), 710–714. 10.11817/j.issn.1672-7347.2015.07.002 26267681

[B7] Cui M. Lu X-L. Lyu Y-Y. Wang F. Xie X-L. Cheng X-Y. (2021). Knowledge and intake of folic acid to prevent neural tube defects among pregnant women in urban China: A cross-sectional study. BMC pregnancy childbirth 21 (1), 1–10. 10.1016/j.wasman.2020.11.054 34154557 PMC8218380

[B8] Dai C. Fei Y. Li J. Shi Y. Yang X. (2021). A novel review of homocysteine and pregnancy complications. BioMed Res. Int. 2021, 6652231–6652314. 10.1155/2021/6652231 34036101 PMC8121575

[B9] Fang Q. Jiang Y. Liu Z. Zhang Z. Zhang T. (2018). Systematic review and meta-analysis of the associations between maternal methylenetetrahydrofolate reductase polymorphisms and preterm delivery. J. Obstet. Gynaecol. Res. 44 (4), 663–672. 10.1111/jog.13566 29315997

[B10] Group MVSR (1991). Prevention of neural tube defects: Results of the medical research council vitamin study. MRC vitamin study research group. lancet 338 (8760), 131–137. 1677062

[B11] Hao L. Ma J. Stampfer M. J. Ren A. Tian Y. Tang Y. (2003). Geographical, seasonal and gender differences in folate status among Chinese adults. J. Nutr. 133 (11), 3630–3635. 10.1093/jn/133.11.3630 14608086

[B12] Jiang M. Huang S. Yuan J. Ma X. Wu X. Zhuo Z. (2021). Association of <i>MTHFR</i> C677T, <i>MTHFR</i> A1298C and <i>MTRR</i> A66G Polymorphisms with Birth Defects in Southern China. J. Hard Tissue Biol. 30 (3), 297–302. 10.2485/jhtb.30.297

[B13] Kanasaki K. Kumagai A. (2021). The impact of micronutrient deficiency on pregnancy complications and development origin of health and disease. J. Obstet. Gynaecol. Res. 47 (6), 1965–1972. 10.1111/jog.14770 33783077

[B14] Li N. Li Z. Ye R. Liu J. Ren A. (2017). Impact of periconceptional folic acid supplementation on low birth weight and small-for-gestational-age infants in China: A large prospective cohort study. J. Pediatr. 187, 105–113. 10.1016/j.jmbbm.2017.06.028 28545876

[B15] Li N. Liu X. An H. Li Z. Zhang L. Zhang Y. (2021). Folic acid supplements and perinatal mortality in China. Available at SSRN 3974539. 10.3389/fnut.2023.1281971 PMC10800445 38260077

[B16] Li X. Jiang J. Xu M. Xu M. Yang Y. Lu W. (2015). Individualized supplementation of folic acid according to polymorphisms of methylenetetrahydrofolate reductase (MTHFR), methionine synthase reductase (MTRR) reduced pregnant complications. Gynecol. obstetric investigation 79 (2), 107–112. 10.1159/000367656 25634728

[B17] Lyu X. Zhang W. Zhang J. Wei Y. Guo X. Cui S. (2021). Morbidity and maternal and infant outcomes of hypertensive disorder in pregnancy in China in 2018. J. Clin. Hypertens. 23 (6), 1194–1204. 10.1111/jch.14248 PMC8678747 33788388

[B18] Nwogu C. M. Okunade K. S. Adenekan M. A. Sekumade A. I. John-Olabode S. Oluwole A. A. (2020). Association between maternal serum homocysteine concentrations in early pregnancy and adverse pregnancy outcomes. Ann. Afr. Med. 19 (2), 113–118. 10.4103/aam.aam_41_19 32499467 PMC7453941

[B19] Ota K. Takahashi T. Han A. Damvaeba S. Mizunuma H. Kwak-Kim J. (2020). Effects of MTHFR C677T polymorphism on vitamin D, homocysteine and natural killer cell cytotoxicity in women with recurrent pregnancy losses. Hum. Reprod. 35 (6), 1276–1287. 10.1093/humrep/deaa095 32478379

[B20] Pan X. Wang P. Yin X. Liu X. Li D. Li X. (2015). Association between maternal MTHFR polymorphisms and nonsyndromic cleft lip with or without cleft palate in offspring, a meta-analysis based on 15 case-control studies. Int. J. Fertil. Steril. 8 (4), 463–480. 10.22074/ijfs.2015.4186 25780529 PMC4355933

[B21] Parthasarathy S. Soundararajan P. Sakthivelu M. Karuppiah K. M. Velusamy P. Gopinath S. C. (2023). The role of prognostic biomarkers and their implications in early detection of preeclampsia: A systematic review. Process Biochem. 126, 238–251. 10.1016/j.procbio.2023.01.017

[B22] Pepe G. Vanegas O. C. Giusti B. Brunelli T. Marcucci R. Attanasio M. (1998). Heterogeneity in world distribution of the thermolabile C677T mutation in 5, 10-methylenetetrahydrofolate reductase. Am. J. Hum. Genet. 63 (3), 917–920. 10.1086/302015 9718345 PMC1377403

[B23] Rai V. Yadav U. Kumar P. Yadav S. K. Mishra O. P. (2014). Maternal methylenetetrahydrofolate reductase C677T polymorphism and down syndrome risk: A meta-analysis from 34 studies. PLoS One 9 (9), e108552. 10.1371/journal.pone.0108552 25265565 PMC4180743

[B24] Ren A. Zhang L. Hao L. Li Z. Tian Y. Li Z. (2007). Comparison of blood folate levels among pregnant Chinese women in areas with high and low prevalence of neural tube defects. Public health Nutr. 10 (8), 762–768. 10.1017/S1368980007246786 17381897

[B25] Shen Z. Wang Y. Ma S. Zhan Y. Wu S. Feng Y. (2019). Risk factors for preterm birth, low birth weight and small for gestational age: A prospective cohort study. Zhonghua liu xing bing xue za zhi= Zhonghua liuxingbingxue zazhi 40 (9), 1125–1129. 10.3760/cma.j.issn.0254-6450.2019.09.020 31594158

[B26] Stamler J. S. Slivka A. (1996). Biological chemistry of thiols in the vasculature and in vascular-related disease. Nutr. Rev. 54 (1), 1–30. 10.1111/j.1753-4887.1996.tb03770.x 8919695

[B27] Tabatabaei R. S. Fatahi-Meibodi N. Meibodi B. Javaheri A. Abbasi H. Hadadan A. (2022). Association of fetal MTHFR C677T polymorphism with susceptibility to neural tube defects: A systematic review and update meta-analysis. Fetal Pediatr. pathology 41 (2), 225–241. 10.1080/15513815.2020.1775734 32536242

[B28] Tinelli C. Di Pino A. Ficulle E. Marcelli S. Feligioni M. (2019). Hyperhomocysteinemia as a risk factor and potential nutraceutical target for certain pathologies. Front. Nutr. 6, 49. 10.3389/fnut.2019.00049 31069230 PMC6491750

[B29] Van Der Put N. M. Van Straaten H. W. Trijbels F. J. Blom H. J. (2001). Folate, homocysteine and neural tube defects: An overview. Exp. Biol. Med. 226 (4), 243–270. 10.1177/153537020122600402 11368417

[B30] Wang D. He Y. Li Y. Luan D. Yang X. Zhai F. (2011). Dietary patterns and hypertension among Chinese adults: A nationally representative cross-sectional study. BMC public health 11 (1), 925–1010. 10.1186/1471-2458-11-925 22168909 PMC3299712

[B31] Wilcken B. Bamforth F. Li Z. Zhu H. Ritvanen A. Redlund M. (2003). Geographical and ethnic variation of the 677C> T allele of 5, 10 methylenetetrahydrofolate reductase (MTHFR): Findings from over 7000 newborns from 16 areas world wide. J. Med. Genet. 40 (8), 619–625. 10.1136/jmg.40.8.619 12920077 PMC1735571

[B32] Yang B. Liu Y. Li Y. Fan S. Zhi X. Lu X.(2013). Geographical distribution of MTHFR C677T, A1298C and MTRR A66G gene polymorphisms in China: Findings from 15357 adults of han nationality. PloS one 8 (3), e57917. 10.1371/journal.pone.0057917 23472119 PMC3589470

[B33] Zarfeshan Fard Y. Kooshkaki O. Kordi Tammandani D. Anani Sarab G. (2019). Investigation of the association between C677T polymorphism of the MTHFR gene and plasma homocysteine level in recurrent fetal miscarriage. J. Obstetrics Gynaecol. Res. 45 (8), 1442–1447. 10.1111/jog.13989 31172624

[B34] Zhang R. Huo C. Wang X. Dang B. Mu Y. Wang Y. (2018). Two common MTHFR gene polymorphisms (C677T and A1298C) and fetal congenital heart disease risk: An updated meta-analysis with trial sequential analysis. Cell. Physiology Biochem. 45 (6), 2483–2496. 10.1159/000488267 29554656

[B35] Zhu J. Zhang J. Xia H. Ge J. Ye X. Guo B. (2021). Stillbirths in China: A nationwide survey. Bjog 128 (1), 67–76. 10.1111/1471-0528.16458 32770714 PMC7754392

[B36] Zhu Z. Yuan L. Wang J. Li Q. Yang C. Gao X. (2021). Mortality and morbidity of infants born extremely preterm at tertiary medical centers in China from 2010 to 2019. JAMA Netw. open 4 (5), e219382. 10.1001/jamanetworkopen.2021.9382 33974055 PMC8114138

