# Supplementary material for: Regional distribution of the MTHFR C677T polymorphism in Chinese females
Source: Front Genet. 2023 Apr 21;14:1139124. doi: 10.3389/fgene.2023.1139124 (PMC10160643; doi:10.3389/fgene.2023.1139124)
Supplement: Supplementary file 1 [file Table2.docx]

Supplementary Table 2 The causes of maternal death in China in 2020

| area | Gestational hypertension （%） | Medical comorbidities（%） | Other（%） |
| --- | --- | --- | --- |
| China | 8.6 | 26.1 | 65.3 |
| Hebei | 6.0 | 30.0 | 64.0 |
| Shandong | 15.4 | 42.3 | 42.3 |
| Henan | 16.5 | 23.5 | 60.0 |
| Shanxi | 17.2 | 37.9 | 44.9 |
| Liaoning | 9.1 | 22.7 | 68.2 |
| Jilin | 16.7 | 25.0 | 58.3 |
| Inner Mongolia | 8.7 | 30.4 | 60.9 |
| Tianjin | 0.0 | 0.0 | 100.0 |
| Heilongjiang | 22.2 | 44.4 | 33.4 |
| Beijing | 0.0 | 20.0 | 80.0 |
| Shaanxi | 4.0 | 12.0 | 84.0 |
| Anhui | 5.6 | 13.9 | 80.5 |
| Jiangsu | 8.3 | 29.2 | 62.5 |
| Gansu | 0.0 | 16.7 | 83.3 |
| Ningxia | 12.5 | 50.0 | 37.5 |
| Qinghai | 18.8 | 25.0 | 56.2 |
| Shanghai | 0.0 | 75.0 | 25.0 |
| Xinjiang | 25.0 | 7.1 | 67.9 |
| Hubei | 2.2 | 28.3 | 69.5 |
| Zhejiang | 0.0 | 16.7 | 83.3 |
| Sichuan | 13.1 | 27.9 | 59.0 |
| Jiangxi | 3.9 | 3.5 | 92.6 |
| Guizhou | 6.0 | 31.3 | 62.7 |
| Yunnan | 6.7 | 23.3 | 70.0 |
| Hunan | 4.0 | 24.0 | 72.0 |
| Chongqing | 0.0 | 19.1 | 80.9 |
| Fujian | 3.2 | 19.4 | 77.4 |
| Tibet | 19.2 | 23.1 | 57.7 |
| Guangdong | 2.9 | 21.7 | 75.4 |
| Guangxi | 4.4 | 22.2 | 73.4 |
| Hainan | 6.7 | 46.7 | 46.6 |

Supported by the Scientific and Technological of Putian of Fujian Province(2018S3F015)
